# Correlation of elevated level of blood midkine with poor prognostic factors of human neuroblastomas

**DOI:** 10.1038/sj.bjc.6600938

**Published:** 2003-05-13

**Authors:** S Ikematsu, A Nakagawara, Y Nakamura, S Sakuma, K Wakai, T Muramatsu, K Kadomatsu

**Affiliations:** 1 Department of Biochemistry, Nagoya University Graduate School of Medicine, 65 Tsurumai-cho, Showaku, Nagoya 466-8550, Japan; 2 Pharmaceuticals Development Department, Meiji Dairies Co., Odawara 250-0862, Japan; 3 Division of Biochemistry, Chiba Cancer Center Research Institute, Chiba 260-8717, Japan; 4 Cell Signals Inc., Tokyo 101-0035, Japan; 5 Department of Preventive Medicine/Biostatistics and Medical Decision Making, Nagoya University Graduate School of Medicine, Nagoya 466-8550, Japan

**Keywords:** growth factor, midkine, molecular target, neuroblastoma, tumour marker

## Abstract

The heparin-binding growth factor midkine (MK) is the product of a retinoic acid-responsive gene, and is implicated in neuronal survival and differentiation, and carcinogenesis. We previously reported that *MK* mRNA expression is elevated in neuroblastoma specimens at all stages, whereas pleiotrophin, the other member of the MK family, is expressed at high levels in favourable neuroblastomas. As MK is a secretory protein, it can be detected in the blood. Here, we show a significant correlation of the plasma MK level with prognostic factors of neuroblastomas. The plasma MK level was determined in 220 patients with neuroblastomas, and compared with that in children without malignant tumors (*n*=17, <500 pg ml^−1^). The plasma MK level became significantly elevated with advancing stages (stage 1: 445 pg ml^−1^ (median), *n*=73; stage 2: 589, *n*=39; stage 3: 864, *n*=40; stage 4: 1445, *n*=56; and stage 4S: 2439, *n*=12). More importantly, a higher MK level was strongly correlated with poor prognostic factors: over 1 year of age (*P*=0.0299), *MYCN* amplification (*P*<0.0001), low *TrkA* expression (*P*=0.0005), nonmass screening, sporadic neuroblastomas (*P*<0.0001), and diploidy/tetraploidy (*P*=0.0007). Thus, these results demonstrate that the plasma MK level is a good marker for evaluating the progression of neuroblastomas. Moreover, considering the ability of antisense MK oligodeoxyribonucleotide to suppress tumour growth of colorectal carcinoma cells in nude mice, as recently reported, the present study suggests that MK is a possible candidate molecular target for therapy for neuroblastomas.

Midkine (MK) is a heparin-binding growth factor that was originally discovered as the product of a retinoic acid-responsive gene during the differentiation of embryonal carcinoma cells (Kadomatsu *et al*, 1988; Tomomura *et al*, 1990; Muramatsu, 2002). The MK family consists of only two members, namely MK and pleiotrophin (PTN; also called HB-GAM), and is distinct from other heparin-binding growth factor families (Kurtz *et al*, 1995; Rauvala *et al*, 2000; Deuel *et al*, 2002; Muramatsu, 2002). Regarding the biological roles of MK, at least three important issues should be pointed out. First, a pivotal role of MK in the migration of inflammatory cells has been revealed by studies involving MK knockout mice; MK-deficient mice are more resistant to vascular restenosis and nephritis induced by reperfusion (Horiba *et al*, 2000; Sato *et al*, 2001). Second, MK exhibits neuroprotective activity (Michikawa *et al*, 1993; Unoki *et al*, 1994; Owada *et al*, 1999), and enhances neurite extension (Muramatsu *et al*, 1993). Induction of MK expression has been detected in reactive astrocytes in ischaemic lesions in human and animal brains (Wang *et al*, 1998; Wada *et al*, 2002). MK is deposited at senile plaques and neurofibrillary tangles in Alzheimer's patients (Yasuhara *et al*, 1993). MK binds to A*β* and inhibits its cytotoxicity (Yu *et al*, 1998). Third, MK is involved in carcinogenesis. Its expression is induced as early as at the precancerous stages of human colorectal and prostate carcinomas (Konishi *et al*, 1999; Ye *et al*, 1999), increases with the stages of human carcinomas, and is significantly linked to the prognosis (O'Brien *et al*, 1996; Mishima *et al*, 1997). MK transforms NIH3T3 cells (Kadomatsu *et al*, 1997), enhances fibrinolysis (Kojima *et al*, 1995), and promotes cell growth (Muramatsu and Muramatsu, 1991; Muramatsu *et al*, 1993; Takei *et al*, 2001), cell survival (Qi *et al*, 2000), cell migration (Takada *et al*, 1997; Maeda *et al*, 1999; Horiba *et al*, 2000; Qi *et al*, 2001), and angiogenesis (Choudhuri *et al*, 1997). MK antisense oligodeoxyribonucleotide suppresses tumour progression in nude mice (Takei *et al*, 2001, 2002).

The neuroblastoma is the most common solid malignant tumour in children. However, the molecular mechanisms underlying its pathogenesis and progression remain unclear, although several molecules, such as MYCN, TrkA, and TrkB, that are linked to the prognosis have been revealed (Brodeur *et al*, 1984; Nakagawara *et al*, 1995). This is one of the reasons why satisfactorily efficient therapies have not been established yet. A possible approach regarding such therapies is to seek molecular targets in neuroblastomas. We previously reported that *MK* mRNA expression is elevated in neuroblastoma specimens at all stages (Nakagawara *et al*, 1995). Interestingly, the other MK family member, PTN, is expressed at high levels in favourable neuroblastomas (Nakagawara *et al*, 1995). As MK is a secretory protein, the blood MK level could be a strong tool for monitoring the status of neuroblastomas. This paper demonstrates that an elevated plasma level of MK is indeed significantly correlated with poor prognostic factors. Our results also indicate that MK could be a candidate molecular target for therapy for neuroblastomas.

## MATERIALS AND METHODS

### Enzyme-linked immunoassay for human MK

An enzyme-linked immunoassay (EIA) for human MK was performed as described previously (Ikematsu *et al*, 2000). Briefly, human MK was produced using *Pichia pastoris* GS115 by transfection with a human MK expression vector, which was constructed into pHIL-D4 (Invitrogen, Carlsbad, California, USA). This yeast-produced human MK was used to immunise rabbits and chickens to raise antibodies. The rabbit anti-human MK antibody (50 *μ*l of 5.5 *μ*g ml^−1^ in 50 mM Tris HCl (pH 8.2), 0.15 M NaCl, 0.1% NaN_3_) was coated onto the wells of microtitre plates (Polysorp plates, Nunc, Rochester, New York, USA) for 20 h at room temperature. After washing with 0.05% Tween 20 in PBS, the wells were blocked with 300 *μ*l of 0.1% casein, 0.01% Microcide I (aMReSCO) in PBS for 20 h at 37°C. Plasma samples (10 *μ*l each) were mixed with 100 *μ*l of 50 mM Tris HCl (pH 8.4), 0.5 M KCl, 0.1% casein, 0.5% BSA, 0.01% Microcide I, and 0.1 *μ*g ml^−1^ peroxidase-labelled chicken anti-human MK antibody. Aliquots of 50 *μ*l of this mixture were added to wells prepared as described above, and further subjected to chromogenic detection at OD_450_ using tetramethylbenzidine as the substrate. This EIA system shows linearity from 0 to 4 ng ml^−1^ of MK, and there is no crossreaction with PTN (Ikematsu *et al*, 2000).

### Plasma samples

Plasma samples were obtained from blood collected from out-come patients without malignant tumours (*n*=17) and neuroblastoma patients (*n*=220). The information is summarised in Table 1

**Table 1:** Blood samples

	***n***
*No malignant tumours*	17
<1 year	11
>1 year	6
	
*Neuroblastomas*	220
Stage 1	73
Stage 2	39
Stage 3	40
Stage 4	56
Stage 4S	12
	
*MYCN* amplification −	176
*MYCN* amplification +	21
	
High *TrkA* expression	157
Low *TrkA* expression	53
	
Mass screening	122
Sporadic	82
	
Hyperdiploidy/pentaploidy	91
Diploidy/tetraploidy	119
	
<1 year	154
>1 year	52

Sum from each group number does not necessarily match the total number, because some information is missing for some individuals.

.

### Statistics

For statistical analysis as to stages, the Kruskal–Wallis test was used to evaluate the statistical differences between groups. The Mann–Whitney *U*-test with Bonferroni's correction was used to further evaluate the difference between the two groups. For analysis as to other prognostic factors, the Mann–Whitney *U*-test was used. *P*<0.05 was taken to be statistically significant.

## RESULTS

### The plasma MK level increases with advancing stages

**Figure 1:**
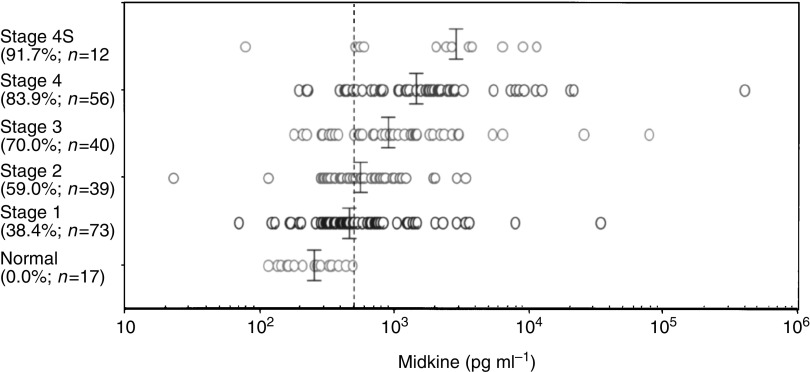
The plasma MK level becomes elevated with advancing neuroblastoma stages. The plasma MK level in children without malignant tumours was 205 pg ml^−1^ (median, *n*=17). Those at stages 1, 2, 3, 4, and 4S were 445 (*n*=73), 589 (*n*=39), 864 (*n*=40), 1445 (*n*=50), and 2439 (*n*=12), respectively. The percentage of cases showing more than the cutoff value (500 pg ml^−1^) is shown in parentheses beneath each stage. The plasma MK level is significantly elevated at all neuroblastoma stages compared with the normal level. Statistical analysis is summarised in Table 2.

Plasma samples were collected from children without malignant tumours and neuroblastoma patients. The information on individuals and neuroblastomas is summarised in Table 1. Plasma from individuals without malignant tumours showed low levels of MK (116–483 pg ml^−1^; median, 205) (Figure 1

). These values are consistent with the data for sera from healthy adults (median, 154 pg ml^−1^; cutoff value, 500 pg ml^−1^) (Ikematsu *et al*, 2000). The EIA employed in the present study showed no difference between serum and plasma (data not shown). These results indicate that the plasma level of MK does not change with age, and that the cutoff value can be set as 500 pg ml^−1^ for children.

The plasma MK level increased with advancing neuroblastoma stages. The median values were 445, 589, 864, 1445, and 2439 pg ml^−1^ for stages 1, 2, 3, 4, and 4S, respectively (Figure 1). The percentages of patients showing MK levels of more than the cutoff value were 38.4, 59.0, 70.0, 83.9, and 91.7% for stages 1, 2, 3, 4, and 4S, respectively (Figure 1). The statistical significance is summarised in Table 2

**Table 2:** Statistical analysis as to stages

**Stage**	**Normal**	**Stage 1**	**Stage 2**	**Stage 3**	**Stage 4**
1	<0.001				
2	<0.001	0.5<			
3	<0.001	0.039	0.5<		
4	<0.001	<0.001	<0.001	0.5<	
4S	0.003	0.001	0.067	0.5<	0.5<
The difference in serum MK level between the six groups was statistically significant (*P*<0.0001, by the Kruskal–Wallis test). *P*-values in this table for differences between the two groups were derived from the Mann–Whitney *U*-test with Bonferroni's correction.					

. Although stage 4S is included in the favourable neuroblastomas along with stages 1 and 2, the MK level was the highest at stage 4S among the stages. This may be interpreted as indicating that one of the parameters influencing the plasma MK level is the tumour volume.

### An elevated plasma MK level is correlated with poor prognostic factors of neuroblastomas

**Figure 2:**
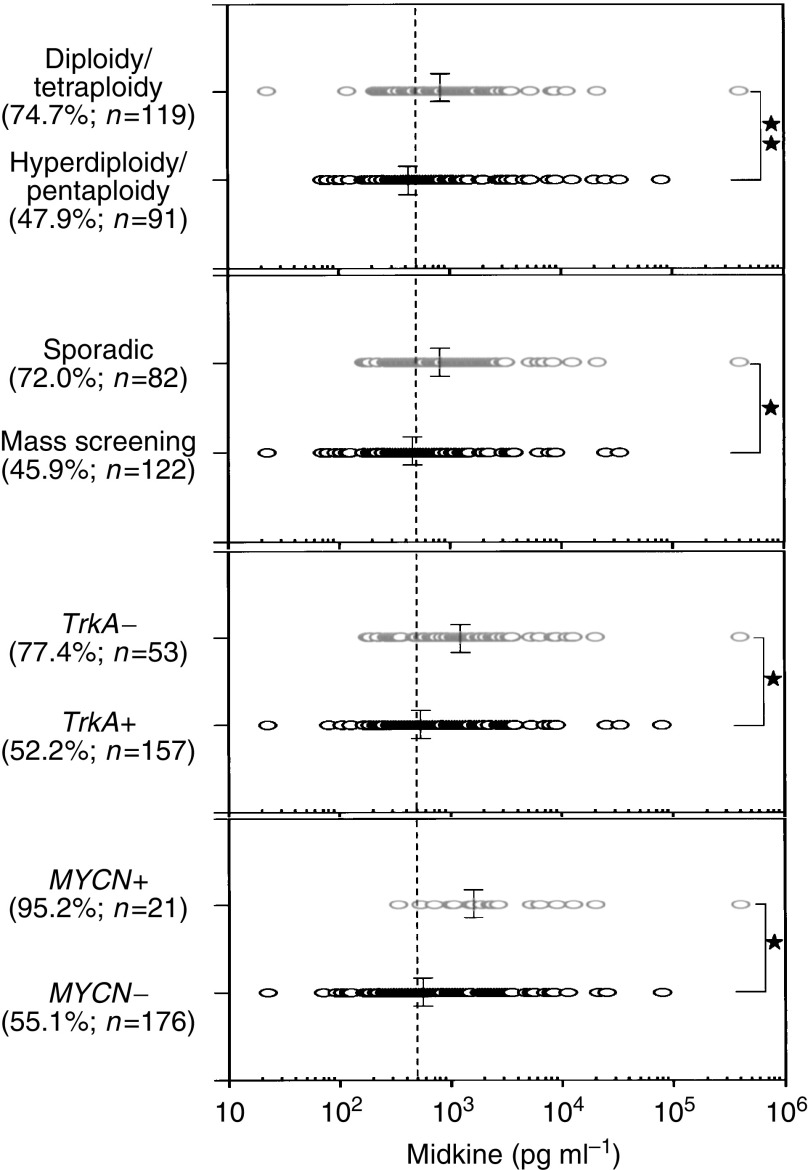
Correlation of the plasma MK level with several prognostic factors of neuroblastomas. Single asterisk, *P*=0.0005 or less; double asterisk, *P*=0.0007. The percentage of cases showing more than the cutoff value (500 pg ml^−1^) is shown in parentheses. Statistical analysis is summarised in Table 3.

The neuroblastoma is one of the best studied tumours. Several factors have been determined to be significantly linked to the prognosis. Cases with amplified *MYCN* show a poor prognosis (Brodeur *et al*, 1984). Low-level expression of the NGF receptor *Trk A* is linked to a poor prognosis (Nakagawara *et al*, 1993). Sporadic neuroblastomas show a worse outcome as compared with neuroblastomas detected by mass screening that is currently being carried out in Japan (Sawada, 1986). Neuroblastomas with diploidy or tetraploidy show a worse prognosis than those with hyperdiploidy or pentaploidy (Look *et al*, 1984). In addition, the outcome in patients of older than 1 year is worse compared with in patients of younger than 1 year (Fortner *et al*, 1968). All these prognostic factors were significantly correlated with a plasma MK level (Figure 2

and Table 3

**Table 3:** Statistical analysis as to several prognostic factors

*MYCN* amplification+ *vs* −	<0.0001
*TrkA* expression low *vs* high	0.0005
Sporadic *vs* mass screening	<0.0001
Diploidy/tetraploidy *vs* hyperdiploidy/pentaploidy	0.0007
1 year< *vs* 1 year>	0.0299

Statistical analysis was performed using the Mann–Whitney *U*-test.

). A higher level of MK was correlated with *MYCN* amplification (*P*<0.0001), low expression of *TrkA* (*P*=0.0005), sporadic neuroblastomas (*P*<0.0001), diploidy/tetraploidy (*P*=0.0007), and older age (*P*=0.0299).

## DISCUSSION

This study demonstrated that the plasma MK level is significantly correlated with prognostic factors of human neuroblastomas. The only exception was that the plasma MK level was the highest at stage 4S among the stages, although stage 4S belongs to the favourable group of neuroblastomas. Stage 4S is a special type of stage 4. Tumours at stage 4S show spontaneous regression (Evans *et al*, 1976). Before spontaneous regression, stage 4S exhibits large tumours and metastasis to the liver, bone marrow, and skin. We previously reported that MK mRNA expression in tumour specimens is very high at all human neuroblastoma stages, including stage 4S, as compared with that in benign tumours, that is, ganglioneuromas (Nakagawara *et al*, 1995). The present data for plasma MK are, therefore, consistent with our previous data for mRNA expression in tumours. Taken together, the results for stage 4S suggest that the tumour volume is one of the factors that influence the plasma MK level. However, the tumour volume may not be the only factor that controls the plasma MK level, because we detected many cases that showed a high MK level even at stage 1 (Figure 1). In favourable neuroblastomas, for example, no *MYCN* amplification was discovered by mass screening, and we often observed high MK plasma levels. It should be noted that, even in favourable neuroblastomas, many cases exhibit poor clinical courses. We can expect that such cases can be evaluated and managed by means of monitoring the plasma level of MK. The importance of the present study is that it provided a chance to follow up prognosis of the patients with several parameters, including the plasma MK level. This perspective study is currently being carried out by our group, which will reveal the biological and clinical significance of an elevated plasma level of MK.

The present results also indicated that MK could be a candidate molecular target for therapy for neuroblastomas, because an elevated plasma MK level is linked with a poor prognosis. Cancer-related activities of MK have been reported by many laboratories. These activities include transforming, migrating, fibrinolytic, mitogenic, antiapoptotic, and angiogenic ones (Kojima *et al*, 1995; Choudhuri *et al*, 1997; Kadomatsu *et al*, 1997; Qi *et al*, 2000, 2001). Furthermore, we recently succeeded in suppressing tumour growth by using antisense MK oligodeoxyribonucleotide (Takei *et al*, 2001, 2002). Thus, ablation of MK production or disruption of its signalling pathway could be a strong means of curing neuroblastomas. Regarding the signalling pathway of MK, receptor-type protein tyrosine phosphatase *ζ*, anaplastic leukaemia kinase, and LDL receptor-related protein (LRP) were recently identified as MK receptors (Maeda *et al*, 1999; Muramatsu *et al*, 2000; Stoica *et al*, 2002). Although it has not been elucidated yet whether or not these receptors form complexes for MK signalling, each protein serves as a receptor transducing intracellular signals for midkine. Further investigation of the MK action mechanism should provide insights as to a therapeutic strategy against aggressive neuroblastomas.

A recent report that 13-*cis*-retinoic acid could be a curative reagent for neuroblastomas (Matthay *et al*, 1999) prompted us to examine the effect of this reagent on MK production, because MK overproduction should make the prognosis worse if 13-*cis*-retinoic acid induced MK production. All-*trans*-retinoic acid as well as 13-*cis*-retinoic acid induced the intracellular production of MK, but did not enhance the secretion of MK, probably because of premature binding, as described below (manuscript in preparation). The endocytosis of MK is completely dependent on LRP, and initiates nuclear targeting by MK, which is partly needed for the antiapoptotic activity of MK (Shibata *et al*, 2002). In addition, LRP functions as a biosynthesis regulator for MK. Overexpression of MK leads to premature binding and aggregation of MK and LRP in the endoplasmic reticulum during the biosynthesis of both proteins, thus preventing MK overproduction that might cause overgrowth or transformation of cells (manuscript in preparation). Taken together, the present results suggest that MK production during tumour development needs enhancing mechanisms at least at two steps: mRNA expression and protein secretion.

One of the characteristics of MK expression is that it is frequently and highly expressed in malignant tumours regardless of the tissue type. This phenomenon is reminiscent of mutations in the p53 gene. We previously reported that an elevated serum MK level was detected in more than 80% frequency of human adult carcinomas (Ikematsu *et al*, 2000). Thus, monitoring of the level of the blood MK is applicable not only to neuroblastomas but also to adult carcinomas. Further assessment of the blood MK level with regard to tumour stages and prognosis of adult malignancy will provide indications for the use of blood MK as a tumour marker for each disease.
